# Caffeic Acid Phenethyl Ester Protects against Amphotericin B Induced Nephrotoxicity in Rat Model

**DOI:** 10.1155/2014/702981

**Published:** 2014-06-16

**Authors:** Atila Altuntaş, H. Ramazan Yılmaz, Ayşegül Altuntaş, Efkan Uz, Murat Demir, Alparslan Gökçimen, Oğuzhan Aksu, Dilek Şenol Bayram, Mehmet Tuğrul Sezer

**Affiliations:** ^1^Department of Nephrology, School of Medicine, Internal Medicine, Süleyman Demirel University, East Campus, 32200 Isparta, Turkey; ^2^Department of Medical Biology, School of Medicine, Mevlana University, 42030 Konya, Turkey; ^3^Department of Dermatology, Isparta State Hospital, 32200 Isparta, Turkey; ^4^Department of Biochemistry, School of Medicine, Süleyman Demirel University, 32200 Isparta, Turkey; ^5^Department of Histology, School of Medicine, Süleyman Demirel University, 32200 Isparta, Turkey; ^6^Department of Endocrinology and Metabolism, School of Medicine, Internal Medicine, Süleyman Demirel University, 32200 Isparta, Turkey

## Abstract

The present study was conducted to investigate whether caffeic acid phenethyl ester (CAPE), an active component of propolis extract, has a protective effect on amphotericin B induced nephrotoxicity in rat models. Male Wistar-Albino rats were randomly divided into four groups: (I) control group (*n* = 10), (II) CAPE group (*n* = 9) which received 10 *μ*mol/kg CAPE intraperitoneally (i.p.), (III) amphotericin B group (*n* = 7) which received one dose of 50 mg/kg amphotericin B, and (IV) amphotericin B plus CAPE group (*n* = 7) which received 10 *μ*mol/kg CAPE i.p. and one dose of 50 mg/kg amphotericin B. The left kidney was evaluated histopathologically for nephrotoxicity. Levels of malondialdehyde (MDA), nitric oxide (NO), enzyme activities including catalase (CAT), and superoxide dismutase (SOD) were measured in the right kidney. Histopathological damage was prominent in the amphotericin B group compared to controls, and the severity of damage was lowered by CAPE administration. The activity of SOD, MDA, and NO levels increased and catalase activity decreased in the amphotericin B group compared to the control group (*P* = 0.0001, *P* = 0.003, *P* = 0.0001, and *P* = 0.0001, resp.). Amphotericin B plus CAPE treatment caused a significant decrease in MDA, NO levels, and SOD activity (*P* = 0.04, *P* = 0.02, and *P* = 0.0001, resp.) and caused an increase in CAT activity compared with amphotericin B treatment alone (*P* = 0.005). CAPE treatment seems to be an effective adjuvant agent for the prevention of amphotericin B nephrotoxicity in rat models.

## 1. Introduction

Amphotericin B (AmB) is widely used for the treatment of fungal infections, especially for candidiasis. It has a toxic effect on fungal cells by making complexes with membrane sterols that can act as transmembrane channels [1–3]. However, its usage is limited due to dose-dependent side effects, commonly nephrotoxicity which may lead to renal failure. In order to prevent its toxicity, new formulations of amphotericin have been developed (liposomal, emulsion, and nanoparticle forms) [1]. Despite the development of pharmaceutical forms of AmB, it has not been able to avoid nephrotoxicity, and the main strategies depend on protective agents [4–6].

Caffeic acid phenethyl ester (CAPE), a flavonoid-like compound, is one of the major components of honeybee propolis [7]. CAPE has no potentially harmful effects on normal cells [8] and has several biological and pharmacological properties, including antioxidant [9, 10], anti-inflammatory [11], anticarcinogenic [12], antiviral [13], and immunomodulatory activities [14]. CAPE has been found to protect rats against carbon tetrachloride-induced liver [15] and kidney injuries [16] and against cisplatin-induced hepatic oxidative damage [17].

Although the protective effects of CAPE against toxic agents have been reported in experimental studies, the role of oxidative stress and the role of CAPE as an antioxidant agent against AmB nephrotoxicity remain unclear. Therefore, this study aims to investigate the potential protective effects of CAPE administration in AmB-induced nephrotoxicity and the possible mechanisms underlying such effects in an experimental model.

## 2. Materials and Method

### 2.1. Animal Model

A total of 40 male Wistar-Albino rats (weighting 200 to 250 g) obtained from the Suleyman Demirel University Animal Production Laboratory were used in the study. All animals were kept in individual cages in a controlled room (22°C, 12 h light/dark cycle) for one week before the start of the experiment. The rats were fed* ad libitum* with standard rat food (Hasyem Ltd., Isparta, Turkey) and tap water. All animals received humane care in compliance with the present institutional guidelines.

### 2.2. Study Design

The rats were randomly divided into four groups (each animal placed separately in a stainless-steel cage) as follows. Group I, the control group (*n* = 10), received 10 mL/kg/day normal saline, intraperitoneally (i.p.) during study; Group II, the CAPE group (*n* = 10), received 10 *μ*mol/kg CAPE (Sigma-Aldrich, St. Louis, MO, USA) i.p. during study [18]; Group III, the AmB group (*n* = 10), received 10 mL/kg/day normal saline i.p. during study and 50 mg/kg AmB (Fungizone, Bristol-Myers Squibb SA, France) once on the second day [19]; Group IV, the AmB plus CAPE group (*n* = 10), received 10 *μ*mol/kg CAPE i.p. during study and 50 mg/kg AmB once on the second day.

### 2.3. Sacrifice of Animals

One hour after the last injection, the rats were anaesthetized with 80 mg/kg i.p. ketamine (Alfamin, Alfasan IBS) and 10 mg/kg xylazine (Alfamin, Alfasan IBS); blood samples were taken from the vena porta, and both kidneys were removed. Left kidneys were stored in 10% formalin solution for histopathological evaluation, and the right kidneys were stored (−80°C) for biochemical analyses. Measurement of serum urea and creatinine was done by Abbott Aeroset.

### 2.4. Tissue Homogenizations and Protein Determination

All kidneys were washed twice with ice-cold saline solution and stored at −80°C until processed. Kidney slices were homogenized (1 : 10, w/v) in ice-cold phosphate buffer (pH 7) containing 0.2 mM Tris-HCl, using a glass Teflon homogenizer. The homogenate was centrifuged at 5.000 rpm for 30 min to remove debris. The supernatant was used for the analyses of selected biochemical parameters. The whole procedure was carried out at +4°C. Protein concentrations of supernatants were measured by the Lowry et al. method [20] with bovine serum albumin as a standard.

### 2.5. Histopathological Examination of the Kidney

For light microscopic evaluation, portions of each kidney were sectioned, fixed in 10% neutral phosphate-buffered formaldehyde, and embedded in paraffin. These specimens were cut into 6 *μ*m thick sections, stained with haematoxylin and eosin, and examined blindly by a pathologist who was unaware of the treatment regimens used. Light microscopy (Olympus BH-2, Tokyo, Japan) was used to evaluate semiquantitative analysis of the kidney sections. The kidneys were examined for tubular epithelial alterations (dilatation, desquamation, vacuolization, necrosis, and atrophy), interstitial inflammation, oedema, and glomerular alterations. All histopathological parameters were graded as follows: (0), no changes; (1) mild, showing cell necrosis, slight degenerative changes, few foci of dilatation, casts, inflammatory infiltration, and oedema; (2) moderate for all changes at different foci throughout the kidney; and (3) severe, extensive, and marked changes [21].

### 2.6. Determination of Malondialdehyde

Malondialdehyde (MDA) levels, an indicator of free radical generation which increases at the end of the lipid peroxidation, were estimated by the double heating method used by Draper and Hadley [22]. This method employs the spectrophotometric measurement of the color generated by the reaction of thiobarbituric acid (TBA) with MDA. For this purpose, 2.5 mL of 100 g/L trichloroacetic acid solution was added to 0.5 mL supernatant in each centrifuge tube, and the tubes were placed in a boiling water bath for 15 min. After cooling in tap water, the tubes were centrifuged at 1000 g for 10 min, and 2 mL of the supernatant was added to 1 mL of 6.7 g/L TBA solution in a test tube; the tube was placed in a boiling water bath for 15 min. The solution was then cooled in tap water, and its absorbance was measured using a spectrophotometer (Shimadzu UV-1601, Japan) at 532 nm. The concentration of MDA was calculated by the absorbance coefficient of the MDA-TBA complex is expressed as nanomoles per gram protein.

### 2.7. Determination of Superoxide Dismutase Activity

Total (Cu-Zn and Mn) superoxide dismutase (SOD) activity was determined according to the method of Sun et al. [23]. The method is based on the inhibition of nitroblue tetrazolium (NBT) reduction by the xanthine/xanthine oxidase system as a superoxide generator. Activity was assessed in the ethanol phase of the supernatant after 1.0 mL ethanol/chloroform mixture (5/3, v/v) was added to the same volume of the sample and centrifuged. One unit of SOD was defined as the enzyme amount causing 50% inhibition in the NBT reduction rate. Activity was expressed as units per milligram protein.

### 2.8. Determination of Catalase Activity

Catalase (CAT) activity was measured according to the method of Aebi [24]. The principle of the assay is based on the determination of the rate constant *k* (dimension: s^−1^, *k*) of hydrogen peroxide decomposition. By measuring the absorbance change per minute, the rate constant of the enzyme was determined. Activities were expressed as *k* (rate constant) per gram protein.

### 2.9. Nitric Oxide Determination

As nitric oxide (NO) measurement is very difficult in biological specimens, tissue nitrite (NO_2_^−^) and nitrate (NO_3_^−^) were estimated as an index of NO production. The method for kidney nitrite and nitrate levels was based on the Griess reaction. Total nitrite (nitrite + nitrate) was measured by spectrophotometry at 545 nm (Ultraspec Plus, Pharmacia LKB Biochrom Ltd., England) after conversion of nitrate to nitrite by copperized cadmium granules. Results were expressed as micromole per gram kidney protein in kidney tissue [25].

### 2.10. Statistical Analysis

Data are presented as means ± standard deviation (S.D.). A computer program (SPSS 9.05, SPSS Inc., Chicago, IL, USA) was used for statistical analysis. The biochemical data were calculated by using Shapiro-Wilk test whether they are of the normal distribution. The test results showed that the calculation was in the standard norm. The one-way analysis of variance (ANOVA) and post hoc multiple comparison tests (LSD) were performed on the data of biochemical variables to examine the difference among groups. A *P* value of < 0.05 is considered as statistically significant.

## 3. Results

In this experimental study, six rats, three from the AmB group and three from the AmB + CAPE group, died before the induction of anesthesia. Autopsy was carried out for all of those rats. No bleeding or lesion was observed in the organs. During homogenization of tissues, a tube which belonged to CAPE rat group was broken; therefore, that rat was also excluded from the study.

### 3.1. Histopathological Changes

**Figure 1:**
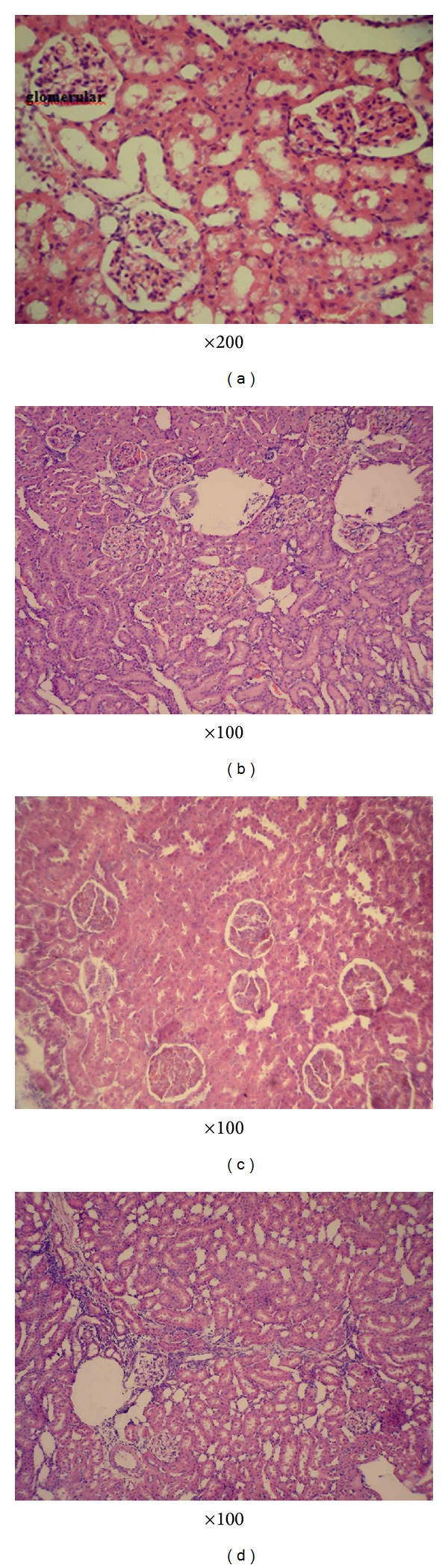
Four micrographs taken from the cortex of kidney in control (a), CAPE (b), Amphotericin B (c), and Amphotericin B + CAPE (d) groups. Control rats show no abnormality (a) and CAPE group had similar findings with the control group (b). Necrotic areas located in the cortex, dilatation-degeneration of the proximal and distal tubules, and proximal and distal epithelial cells which are abundant hyperchromatic nuclei are clearly observed in the rat kidneys given amphotericin B (c). Although CAPE treatment reduced the severity of renal damage, it was not able to protect completely the histopathological damages (d).

As shown in figures and Table 1, renal histopathology of controls and the CAPE group was normal (Figures 1(a) and 1(b)). When compared with controls, prominent histopathological changes such as proximal and distal tubular dilatation, hyperchromatic cell nuclei in tubular epithelial cells, hemorrhages in the medullar area, necrosis in cortical areas, mononuclear cell infiltrations in both the medullar and cortical areas, and dilatations/degenerations in the collecting ducts were observed in the rats given amphotericin B (Figure 1(c)). Although the administration of CAPE mitigated histopathological damage in the nephrotoxicity caused by AmB, histological damage was not completely recovered as seen in controls (Figure 1(d)).

### 3.2. Tissue MDA, NO, SOD, and Catalase Activities

**Figure 2:**
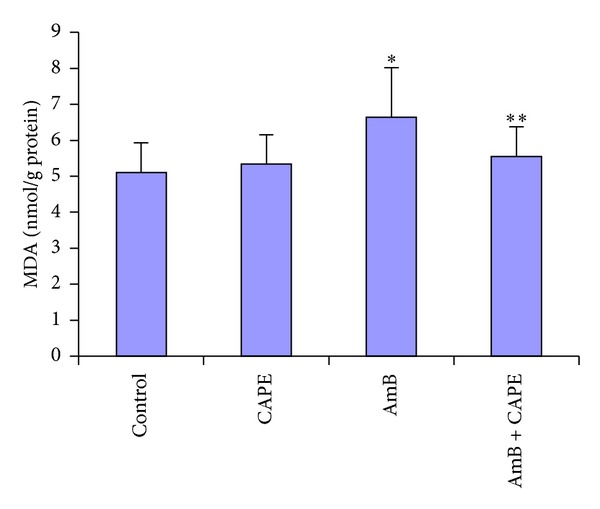
Malondialdehyde (MDA) levels in kidney tissues of rats in control, CAPE, AmB, and AmB + CAPE subgroups (CAPE, caffeic acid phenethyl ester; AmB, Amphotericin B). *P = 0.003 and **P > 0.05 compared with control group.

**Figure 3:**
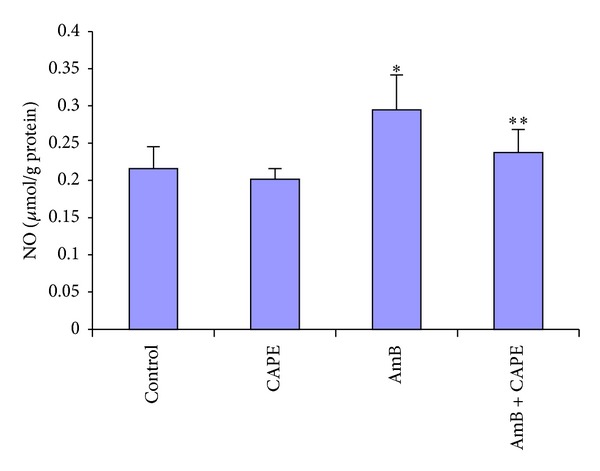
Nitric oxide (NO) levels in kidney tissues of rats in control, CAPE, AmB, and AmB + CAPE subgroups (CAPE, caffeic acid phenethyl ester; AmB, Amphotericin B). *P < 0.0001 and **P > 0.05 compared with control group.

All measured enzyme activities were similar between control and CAPE groups. In the AmB-treated rats, the kidney MDA and NO levels were significantly higher than those of the control group (6.64 ± 1.38 versus 5.10 ± 0.83 nmol/g protein; 0.295 ± 0.04 versus 0.216 ± 0.03 *μ*mol/g protein, resp.) (*P* = 0.003 and *P* < 0.0001, Figures 2 and 3). CAPE administration with AmB attenuated increases in MDA and NO generation (*P* = 0.044 and *P* = 0.002, resp.) in the kidney compared to AmB alone. However, MDA and NO levels were not significantly different between the AmB + CAPE group and the control group.

**Figure 4:**
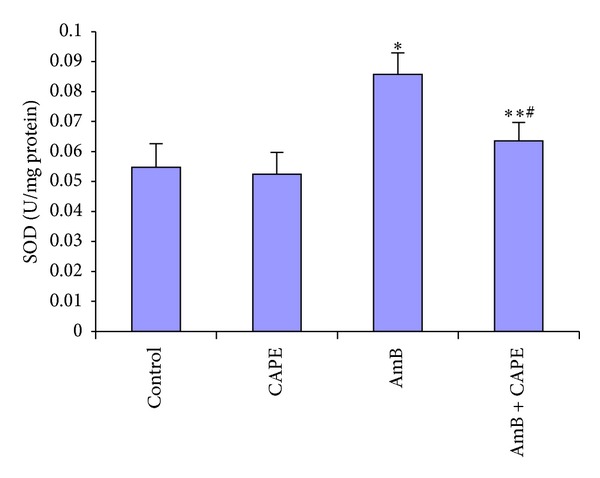
Superoxide dismutase (SOD) activities in kidney tissues of rats in control, CAPE, AmB, and AmB + CAPE subgroups (CAPE, caffeic acid phenethyl ester; AmB, Amphotericin B). *P < 0.0001 and **P = 0.023 compared with control group. ^#^ *P* < 0.0001 compared with AmB group.

In the AmB group, SOD activity significantly increased according to the control group (0.086 ± 0.007 versus 0.055 ± 0.008 U/mg protein, resp.) (*P* < 0.0001, Figure 4). CAPE administration with AmB injection suppressed the increase in SOD activity compared with AmB alone (0.063 ± 0.006 versus 0.086 ± 0.007 U/mg protein, *P* < 0.0001); however, in the AmB + CAPE group, kidney SOD activity was significantly higher than those of the control group (*P* = 0.02).

**Figure 5:**
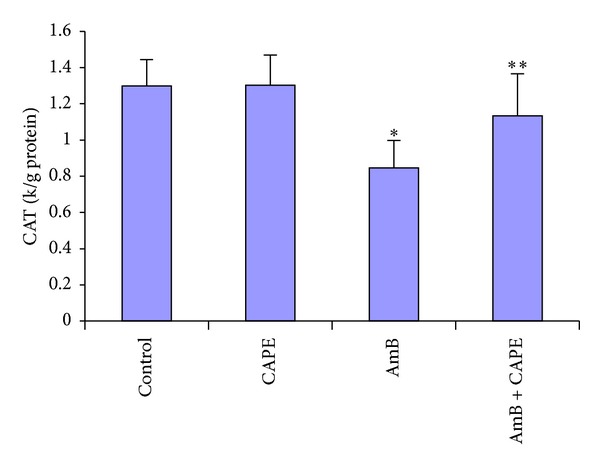
Catalase (CAT) activities in kidney tissues of rats in control, CAPE, AmB, and AmB + CAPE subgroups (CAPE, caffeic acid phenethyl ester; AmB, Amphotericin B). *P < 0.0001 and **P > 0.05 compared with control group.

The CAT activity in the kidney was significantly reduced in the AmB group compared with the control group (0.85 ± 0.15 versus 1.30 ± 0.15 k/g protein) (*P* < 0.0001, Figure 5). However, CAT activity was higher in the AmB + CAPE group compared to the AmB group (1.13 ± 0.23 versus 0.85 ± 0.15 k/g protein resp., *P* = 0.005). There was no significant difference in the activity of CAT between the AmB + CAPE and the control groups.

### 3.3. Urea and Creatinine

Similar to a previous study [19], AmB administration at a dose of 50 mg/kg resulted in acute renal failure. As shown in Table 2, serum BUN and creatinin levels of AmB and AmB plus CAPE groups were significantly higher than the control group (resp.; *P* = 0.0001, *P* = 0.0001). However, there was no statistically significant difference between AmB and AmB plus CAPE groups in terms of the levels of BUN and serum creatinine (*P* = 0.22 and *P* = 0.83, resp.).

## 4. Discussion

The important finding of this study is that a single dose of AmB (50 mg/kg) resulted in prominent nephrotoxicity as shown by histopathological examination and biochemical markers. Elevation of lipid peroxidation product MDA, NO, and SOD activity with reduction of antioxidant enzyme catalase in renal tissue following AmB administration shows that oxidative stress with respect to free-radical damage is one of the possible mechanisms in the pathophysiology of AmB nephrotoxicity. In addition, giving CAPE to the rats at a dose of 10 *μ*mol/kg showed a renoprotective effect on AmB induced tissue damage.

Histopathologic damage occurred after AmB administration and was more prominent in AmB group compared to AmB plus CAPE group. CAPE administration has a beneficial effect on histopathologic damage. This beneficial effect perhaps accomplishes due to the decrease in oxidative stress via CAPE administration. Nephrotoxicity, the most serious adverse effect of AmB, includes decrease of the glomerular filtration rate (GFR), renal tubular acidosis, and antidiuretic hormone- (ADH-) resistant polyuria [26]. Moreover, AmB forms pores in membranes that cause tubular dysfunction [2]. Feldman et al. showed that prophylactic NAC, an antioxidant, prevented tubular necrosis and decreases in the GFR that is related to high doses [19]. Odabasi et al. demonstrated that NAC reduced AmB induced renal tubular apoptosis [27]. However, discordance in serum creatinine level and the severity of histopathologic damage between AmB and AmB plus CAPE groups could be explained by different possible mechanisms. First, CAPE, an antioxidant, could not improve all possible AmB-induced nephrotoxicity mechanisms such as decrease in blood flow rate and tubular toxicity. Second, it may be related to CAPE dose or duration of usage. In additıon, as a result of our study we believe that the rats which died in the groups of AmB and AmB plus CAPE categorization, the AmB nephrotoxicity played a significant role and caused their death. The reason for the inability of CAPE to prevent their death may be caused by the fact that the dosage of CAPE has been insufficient.

Oxidative stress is related to the production of free radicals and reactive oxygen species (ROS). There are a plenty of antioxidant systems against excessive amounts of ROS produced oxidative stress [28]. Initially, the SOD detoxifies the superoxide anion into hydrogen peroxide [29]. Then, the enzymes, CAT and glutathione peroxidase (GSH-Px), try to produce water by converting hydrogen peroxide to prevent ROS injury in the cell. The glutathione is an important source during that process for GSH-Px activity. The exogenous antioxidant molecules can assist for the detoxification of ROS, if the endogen antioxidant system fails [28]. Pekmez et al. and Serarslan et al. suggested that the increased levels of SOD in their research of nephrotoxicity modellings in rats [30, 31]. In this study, administration of AmB increased both nitrous oxide and SOD. Increases in SOD enzyme activity correspond with enhanced resistance to oxidative stress [32]. This could be related with excessive ROS production and stimulation of SOD activity. SOD activity was lower in the AmB plus CAPE group compared to AmB group. This finding shows that CAPE significantly prevents the elevation of kidney tissue SOD activity by scavenging free radicals produced by AmB. The activity of CAT enzyme in kidney tissues from rats decreased significantly after AmB administration. The decreased CAT activity in renal tissue of the rats which were administered AmB may result from the overconsumption of this enzyme associated with the increased oxidative stress. The reduction of CAT activity in renal tissue of animals treated with AmB alone was prevented by cotreatment with CAPE. Oktem et al. and Meydan et al. previously reported that CAPE caused an elevation in CAT in drug-induced nephrotoxicity models of rat kidneys [33, 34]. The exact mechanism of CAPE on enzyme activities is discovered yet; nevertheless, the transcriptional and/or translational pathways of these antioxidant enzymes can be changed by CAPE.

Aygün et al. discovered that NO level in the kidney tissue was multiplied by the gentamicin management, and CAPE prevented this increase at a statistically significant level [35]. Similarly, Uz et al. showed marked elevation in NO level in the damaged kidney tissue of the methotrexate-treated rats, and therefore this increment attenuated significantly via CAPE [36]. NO has an important role in modulating oxidant stress and tissue damage. In this study, we found that NO concentration was higher in the AmB group compared to the control and AmB plus CAPE groups. Increased NO concentration could be related with the induction of inducible nitric oxide synthase, which is leading to generation of ONOO and nitrosative stress [37]. The decrease in NO concentration after the administration of CAPE in the AmB plus CAPE group compared to the AmB group suggests that CAPE has a positive effect on nitrosative stress via the decrease in the generation of the inducible nitric oxide synthase enzymes.

Lipids, the main component of the plasma membrane, are susceptible to the oxidation of ROS. Peroxidation of lipids results in aldehyde release, including MDA, which is widely used for the assessment of lipid peroxidation [38, 39]. In our study, we found that AmB caused MDA generation. Administration of CAPE reduced MDA levels significantly in the AmB plus CAPE group. This result concludes that the generation of free radicals and subsequent lipid peroxidation may play a role in AmB nephrotoxicity and CAPE could be an effective agent for the reduction of AmB-induced oxidative stress and lipid peroxidation. Oktem et al. found that the injection of methotrexate increased MDA levels in renal tissue of rats cotreated with CAPE and that caused a significant decrease in the levels of MDA in renal tissue [40]. Ginis et al. demonstrated that ifosfamide induced brain damage increased MDA levels in rat and CAPE administration significantly reduced MDA levels [41]. We propose that CAPE plays an important role in kidney as a potent collector of free radicals to prevent the toxic effects of AmB.

In the current study, we found that antioxidant enzyme SOD activity increased but catalase activity decreased after AmB application. However, compared to the AmB group, SOD activity decreased and catalase activity increased after the administration of CAPE in the AmB plus CAPE group. It is possible that AmB causes an excessive production of free oxygen radicals, generating O_2_^−^ and/or possible induction of SOD activity [21]. Furthermore, excessive O_2_^−^ scavenges by SOD, so that the cells are protected against the toxic effects of superoxide radicals, reflecting the decrease in catalase enzyme activity. The effect of CAPE administration appears to decrease in SOD activity but increase in catalase activity. This could be associated with the reduction of oxidative stress, nitrosative stress, and the generation of O_2_^−^. Thus, the CAPE treated group is protected against the toxic effect of AmB-induced oxidative stress and reflects a decrease in MDA and SOD activity but an increase in catalase activity.

In conclusion, AmB toxicity remains high despite developments in drug formulations. The main strategies are based on prevention. The role of oxidative stress in AmB toxicity is clear. Therefore, administration of CAPE seems to be an alternative agent for the management of AmB toxicity. Further clinical studies are necessary for confirmation of these positive effects in clinical settings.

## Figures and Tables

**Table 1:** The scores of histological changes of kidney damage in study groups.

Groups	*n*	Proximal and distal tubular dilatation	Hyperchromatic cell nuclei	Hemorrhagic areas in medulla	Necrotic areas in cortex	Mononuclear cell infiltration in medulla	Mononuclear cell infiltration in cortex	Dilatations/ degenerations in collecting ducts
1-Control	10	0.00 ± 0.00	0.30 ± 0.48	0.00 ± 0.00	0.00 ± 0.00	0.00 ± 0.00	0.00 ± 0.00	0.10 ± 0.32
2-CAPE	9	0.11 ± 0.33	0.33 ± 0.71	0.11 ± 0.33	0.00 ± 0.00	0.11 ± 0.33	0.22 ± 0.44	0.22 ± 0.44
3-AmB	7	2.86 ± 0.38	1.57 ± 0.98	1.86 ± 0.69	2.00 ± 0.58	0.86 ± 0.69	2.00 ± 0.58	2.57 ± 0.79
4-AmB + CAPE	7	1.14 ± 1.07	1.00 ± 0.58	0.29 ± 0.49	0.71 ± 0.49	1.14 ± 0.69	0.8571 ± 0.6901	0.71 ± 0.49

*P*-values								
1-2		NS	NS	NS	NS	NS	NS	NS
1–3		0.0001	0.001	0.0001	0.0001	0.001	0.0001	0.0001
1–4		0.0001	0.049	NS	0.0001	0.0001	0.001	0.21
2-3		0.0001	0.001	0.0001	0.0001	0.004	0.0001	0.0001
2–4		0.001	NS	NS	0.0001	0.0001	0.012	NS
3-4		0.0001	NS	0.0001	0.0001	NS	0.0001	0.0001

NS: not significant. Values are expressed as mean ± standard error of mean.

**Table 2:** BUN and serum creatinine levels in rats subjected to amphotericin induced nephrotoxicity.

GROUPS	*n*	BUN (mg/dL)	Creatinine (mg/dL)
1-Control	10	21.4 ± 3.4	0.58 ± 0.10
2-CAPE	9	18.7 ± 2.6	0.51 ± 0.03
3-AmB	7	73.5 ± 39.2	1.61 ± 1.13
4-AmB + CAPE	7	88.5 ± 29.1	1.54 ± 0.74

*P*-values			
1-2		NS	NS
1–3		<0.0001	<0.0001
1–4		<0.0001	=0.005
2-3		<0.0001	<0.0001
2–4		<0.0001	=0.003
3-4		NS	NS

NS: not significant. Values are expressed as mean ± standard error of mean.
